# Consumption of Sugar-Sweetened Beverages Is Associated with Components of the Metabolic Syndrome in Adolescents

**DOI:** 10.3390/nu6052088

**Published:** 2014-05-23

**Authors:** Te-Fu Chan, Wei-Ting Lin, Hsiao-Ling Huang, Chun-Ying Lee, Pei-Wen Wu, Yu-Wen Chiu, Chun-Chi Huang, Sharon Tsai, Chih-Lung Lin, Chien-Hung Lee

**Affiliations:** 1 Graduate Institute of Medicine, College of Medicine and Department of Obstetrics and Gynecology, Kaohsiung Medical University Hospital, Kaohsiung Medical University, 100 Shih-Chuan 1st Road, Kaohsiung 807, Taiwan; E-Mail: tefu.chan@msa.hinet.net; 2 Institute of Environmental and Occupational Health Sciences, National Yang Ming University, 155, Section 2, Li-Nong Street, Taipei 112, Taiwan; E-Mail: wtlin0123@gmail.com; 3 Department of Oral Hygiene, College of Dental Medicine, Kaohsiung Medical University, 100 Shih-Chuan 1st Road, Kaohsiung 807, Taiwan; E-Mail: hhuang@kmu.edu.tw; 4 Department of Public Health, College of Health Sciences, Kaohsiung Medical University, 100 Shih-Chuan 1st Road, Kaohsiung 807, Taiwan; E-Mails: cying@ms19.hinet.net (C.-Y.L.); catstar1211@gmail.com (P.-W.W.); 5 Department of Family Medicine, Kaohsiung Medical University Hospital, Kaohsiung Medical University, 100 Shih-Chuan 1st Road, Kaohsiung 807, Taiwan; 6 Health Policy and Systems Management Program, Health Sciences Center, School of Public Health, Louisiana State University, 433 Bolivar St, New Orleans, LA 70112, USA; E-Mail: YChiu@lsuhsc.edu; 7 Department of Laboratory Medicine, Kaohsiung Municipal Hsiao-Kang Hospital, 482 Shanming Road, Kaohsiung 812, Taiwan; E-Mails: 0870791@kmhk.org.tw (C.-C.H.); 0870718@kmhk.org.tw (S.T.); 8 Department of Surgery, College of Medicine, Kaohsiung Medical University Hospital, Kaohsiung Medical University, 100 Shih-Chuan 1st Road, Kaohsiung 807, Taiwan

**Keywords:** adolescent, metabolic syndrome, obesity, sugar-sweetened beverages

## Abstract

Sugar-sweetened beverages (SSBs) are the principle source of added sugar in diets. Cardiometabolic disturbances can occur from early childhood to adulthood. The aim of this cross-sectional study was to examine the gender-specific association of SSB intake with metabolic syndrome (MetS) and its components among adolescents in Taiwan. A total of 2727 adolescents aged 12 to 16 years randomly selected from three diverse economic areas in Southern Taiwan by using a multistage-sampling strategy participated in this study. Demographic, dietary, physical and anthropometric parameters were measured, and serum lipid profiles and glucose levels were determined. The International Diabetes Federation (IDF) specifies that MetS requires abdominal obesity and ≥2 abnormal components, and Cook criteria for MetS require ≥3 abnormal components. We applied survey-data modules to data analyses, and used multiple regression and logistic models to adjust for covariates. An increased SSB intake was linked to a greater waist circumference in both sexes and to systolic blood pressure in boys (*P* for trend: ≤0.043). Male moderate and high consuming SSB drinkers exhibited triglyceride levels that were 8.0 and 8.2 mg/dL significantly higher, respectively, than those of nondrinkers. Compared with nondrinkers, boys who consumed >500 mL/day (high quantity) of SSBs exhibited 10.3-fold (95% confidence intervals (CIs): 1.2-90.2) and 5.1-fold (95% CIs: 1.01-25.5) risks of contracting MetS, as defined by the IDF and Cook criteria for MetS, respectively. In girls, the risk estimates for the same comparison were not significant by the IDF criteria (6.5-fold risk, 95% CIs: 0.9-∞) or Cook criteria (5.9-fold risk, 95% CIs: 0.8-43.8) for MetS. High SSB consumption was also linked to 1.9-fold (95% CIs: 1.1-3.1) and 2.7-fold (95% CIs: 1.3-5.7) higher risks of being at a greater overall metabolic risk in girls and boys, respectively. In conclusion, a high SSB intake is associated with adolescent MetS among boys but not girls in Taiwan.

## 1. Introduction

Epidemiological evidence has indicated that cardiometabolic disturbances can occur from early childhood to adulthood [1,2,3]. Metabolic syndrome (MetS) is a clustering of three or more cardiometabolic risk factors, including abdominal obesity, elevated blood pressure, elevated fasting plasma glucose (FPG), high serum triglycerides (TG) and low high-density lipoprotein cholesterol (HDL-C) levels [4]. In adults, this metabolic disorder has been determined to induce an approximately 2-fold risk for cardiovascular death, a 3-fold risk for cardiovascular complications and a 5-fold risk for type 2 diabetes [5,6,7,8]. In children, obesity has been associated with the development of MetS [9,10], and in prospective studies, pediatric MetS has been identified as a crucial predictor for adult MetS, type 2 diabetes and cardiovascular disease [11,12]. Adolescents are one of the major groups who consume a high amount of sugar-sweetened beverages (SSBs) [13]; however, limited data are available regarding the effects of SSB consumption among adolescents on pediatric MetS.

SSBs, a liquid form of carbonated or noncarbonated energy, are the principle source of added sugar in diets [14]. In adults, the intake of SSBs has been associated with an enhanced risk of weight gain and of developing obesity and obesity-related disorders, such as MetS, type 2 diabetes, coronary heart disease and stroke [15,16,17]. Recent investigations have indicated that, compared with isoenergetic solid carbohydrates, liquid carbohydrates, especially SSBs, generate less satiety and overall energy intake is increased because of incomplete dietary compensation [18,19,20].

The prevalence of obesity among adolescents in Taiwan has increased by 27.7% in girls and 25.8% in boys in recent decades [21], and has become a critical concern in pediatric health nationwide. One study conducted in Taiwan reported that the consumption of SSBs was associated with a 3.2- to 4.9-fold risk of obesity [2]. The purpose of this study was to investigate the gender-specific association of SSB consumption with MetS and its components among adolescents in the same large-scale cross-sectional survey in Taiwan [2].

## 2. Materials and Methods

### 2.1. Participants and Study Design

This study was conducted in three areas characterized by various economic backgrounds and urbanization levels in Southern Taiwan—Kaohsiung City (urban region), Pingtung County (suburban region) and Taitung County (rural region)—and involved monitoring the Multilevel Risk Profiles for Adolescent Metabolic Syndrome (mRP-aMS). Adolescents aged 12 to 16 years who were included in the entry lists of junior high schools in these areas were the target population. The mRP-aMS protocol was approved by the ethics committee of Kaohsiung Medical University, and all data collection was conducted in accordance with the guidelines for ethical conduct in human research. Informed written consent was obtained from the adolescents and their parents or guardians.

The mRP-aMS, performed between 2007 and 2009, was a cross-sectional survey of a representative sample of adolescents in Grades 7-9 in Southern Taiwan. The study consisted of a school visit at which questionnaire surveys were administered, anthropometric measurements were obtained and blood samples were subsequently collected. The details of the research method were reported elsewhere [2]. Briefly, a multistage, geographically stratified cluster sampling design was used to recruit study participants. A total of 3784 students randomly selected from 36 schools agreed to participate in the questionnaire and anthropometric surveys (97.5% response rate). Among these students, 2727 adolescents (72.1%) participated in the clinical blood examinations.

### 2.2. Data Collection

A structured questionnaire was used to collect research data. Information obtained included demographic factors, personal disease history, lifestyle behaviors, dietary intake and physical activity, as well as cigarette smoking and alcohol consumption status. We used a semiquantitative food-frequency questionnaire containing 23 food groups to assess daily dietary patterns exhibited during the previous month. The intake of various SSBs, including soft drinks, fruit drinks and sweetened teas, was obtained from the responses provided in the completed food-frequency questionnaire. SSB drinkers were defined as adolescents who had consumed at least one serving of any type of SSB per week over the prior month. We calculated the total SSB consumption per day for each participant, and for data analysis, classified SSB consumption as non-intake, 1-500 mL and intake >500 mL according to the standard serving size in Taiwan. The Taiwanese Food and Nutrients Databank was employed to estimate the total calories consumed based on individual food consumption data [22]. Furthermore, weekday and weekend patterns of physical activity, including physical education classes in schools, after-school physical activity, extracurricular activities or training, physical activities in the evenings, spare time and weekend activities and static activities for each participant in a regular week were measured using nine group questions. We transformed the activity data into metabolic equivalent task (MET) measurements and calculated the overall MET-minutes per week [2]. The adolescents were categorized into three groups according to the tertiles of total physical activity.

### 2.3. Anthropometric and Clinical Measurements

Qualified examiners performed anthropometric measurements using a standardized process after collecting the questionnaire data. Anthropometric indicators comprised height, weight, hip circumference (HC) and waist circumference (WC), body fat, systolic blood pressure (SBP) and diastolic blood pressure (DBP). Body fat percentage was determined using a body impedance system (BF-800, Tanita Corp, Tokyo, Japan), and body adiposity index (BAI, %), another indicator for human body fat, was calculated as [(hip in cm)/(height in m)^1.5^] - 18 [23]. The formula (weight in kg)/(height in m)^2^ was used to compute body mass index (BMI) [24].

We collected blood samples from the participants after 3 weeks of data collection. Clinical specimens were obtained in the morning by conducting venipuncture after the participants underwent a 10-h overnight fast at the health center of each school. The lipid profiles, including HDL-C, low-density lipoprotein cholesterol (LDL-C), TGs and total cholesterol levels, as well as FPG levels, were enzymatically quantified by employing a chemistry autoanalyzer using commercially available reagents (TBA-c16000 automatic analyzer, Toshiba, Tokyo, Japan) [25].

### 2.4. Definitions of Metabolic Syndrome

Because metabolic and anthropometric parameters change with age and pubertal growth, applying the adult MetS definition to adolescents is problematic [7]. In this study, we measured adolescent MetS by using the International Diabetes Federation (IDF) consensus criteria [7] and the Cook *et al*. criteria for MetS, which involves considering age, sex and height for several MetS components [26]. In the IDF criteria, a diagnosis of pediatric MetS requires abdominal obesity (WC ≥ 90th percentile or adult cutoff if lower) and the presence of two or more of the following clinical features: low HDL-C (<40 mg/dL), elevated TGs (≥150 mg/dL), increased FPG (≥100 mg/dL or known type 2 diabetes) and high blood pressure (SBP ≥ 130 mmHg or DBP ≥ 85 mmHg). According to Cook *et al*. criteria for MetS [26], a diagnosis of adolescent MetS requires at least three of the following risk components: abdominal obesity (WC ≥ 90th age- and sex-specific percentile), low HDL-C (≤40 mg/dL), elevated TGs (≥110 mg/dL), increased FPG (≥110 mg/dL) and high blood pressure (blood pressure ≥90th age-, sex- and height-specific percentile).

### 2.5. Statistical Analysis

The data were first arranged based on computed complex sampling weights. Stata v13 survey-data statistical modules (StataCorp., College Station, TX, USA) were applied to accommodate the complex sampling design. We used multivariate linear regression models to assess the association between SSB intake and cardiometabolic risk factors. Multivariate-adjusted mean and regression coefficients were employed to determine the effect of diverse levels of SSB intake on continuous outcomes. A fundamental regression model including age, sex and study area was used to assess potential confounders. Factors that altered the effect of interest by >10% or that had been determined as confounders in prior studies were considered confounding factors [27,28]. Covariates, including study area, age, physical activity, total calories, the intake of meat, fruit, fried food, food with jelly/honey, alcohol consumption and cigarette smoking, were evaluated as confounders.

Because the IDF diagnostic definition for pediatric MetS is an interim set of criteria, a range of cutoff values principally based on the Adult Treatment Panel III adult criteria for classifying MetS components have been used in previous studies [7]. To assist in the evaluation of IDF and Cook *et al*. criteria for MetS [7,26], a two-step cluster analysis was conducted to identify adolescents at low, median and high metabolic risk; this analysis method has been applied in previous studies [3,29]. This statistical method was used to determine the nature clusters of subjects, in which the subjects in the same cluster are more similar to each other than they are to subjects in other clusters. Because MetS is a cluster of risk factors that occur jointly, cluster analysis is an appropriate technique for identifying high-risk clusters [29]. In the first cluster step, we employed an agglomerative hierarchical clustering procedure, in which each observation was first considered as a separate group, the closest two groups were then combined, and this process was continued until all observations merged with progress up the hierarchy. The *k*-means partition cluster method was employed as the second cluster step. Subclusters and seed values generated from the first cluster step were used as inputs, and an iterative process, in which each observation was assigned to subclusters whose mean was closest, was performed. These steps continued until no changes were observed in subclusters. Standardized components of MetS, including WC, HDL-C, TG, FPG, SBP and DBP levels, were used to identify low, median and high overall metabolic risk clusters for girls and boys, respectively. Furthermore, to evaluate the effect of SSB intake on binary outcomes for the MetS components and trinary outcomes for the BMI and metabolic risk clusters, we employed the adjusted odds ratio (aOR) and 95% confidence intervals (CIs), which were respectively derived from binary and polytomous logistic regression models, to estimate the associated risks [30].

## 3. Results

Table 1 shows the distribution of demographic factors, physical activity and energy intake associated with the intake of SSBs among adolescents. After adjusting for the complex sampling design, we determined that a higher proportion of boys than girls consumed a high amount of SSBs (>500 mL; 32.1% *vs*. 18.5%). Adolescents who ingested a high amount of SSBs typically exhibited an increased total calorie intake (*P*_trend_ < 0.001 for both sexes). Among the girls, discrepancies in the SSB intake pattern were noted across various economic areas.

Table 2 shows the multivariate-adjusted means and regression coefficients of the cardiometabolic risk factors associated with SSB intake. An increased consumption of SSBs was linked to a greater WC, with a significantly increased WC observed in girls who exhibited a >500 mL/day intake and in boys who exhibited SSB intake. Compared with nondrinkers, male median (1-500 mL/day) and high (>500 mL/day) SSB drinkers had an 8.0 and 8.2 mg/dL greater concentration of TGs (78.4 and 78.6 *vs*. 70.4 mg/dL), respectively. A higher level of SSB consumption was related to a higher level of SBP in boys (adjusted β = 0.6 mmHg, *P*_trend_ = 0.043). In both girls and boys, a notable dose-dependent effect of SSB intake on HC, body fat, BAI and BMI was observed.

Table 3 shows the prevalence rate and risks of developing MetS and its components among adolescents according to the IDF and Cook *et al*. criteria for MetS. The prevalence rate of IDF MetS was 1.1% and 2.1% for girls with a median and high amount of SSB consumption, and 3.5% and 5.4% for boys, respectively, with a large WC being the major contributor of MetS (prevalence rates were 13.4% and 22.7% for median and high intake in girls, and 12.1% and 18.7% in boys, respectively). Compared with nondrinkers, boys who consumed >500 mL of SSBs per day exhibited a 10.3-fold risk (95% CIs: 1.2-90.2) of developing IDF MetS, and girls had a non-significant 6.5-fold risk (95% CIs: 0.9-∞) of developing MetS. Alternatively, the prevalence rate of MetS according to Cook criteria was 1.9% and 3.4% for median and high SSB intake in girls, and 4.1% and 5.1% in boys, respectively, with raised blood pressure being the main contributor of MetS (prevalence rates were 16.2% and 23.0% for median and high intake in girls, and 17.3% and 22.0% in boys, respectively). Compared with nondrinkers, boys who consumed >500 mL/day of SSBs exhibited a 5.1-fold (95% CIs: 1.01-25.5) risk of being diagnosed with Cook criteria for MetS, and girls who ingested >500 mL of SSBs per day had a non-significant 5.9-fold risk (95% CIs: 0.8-43.8) of developing MetS.

**Figure 1:**
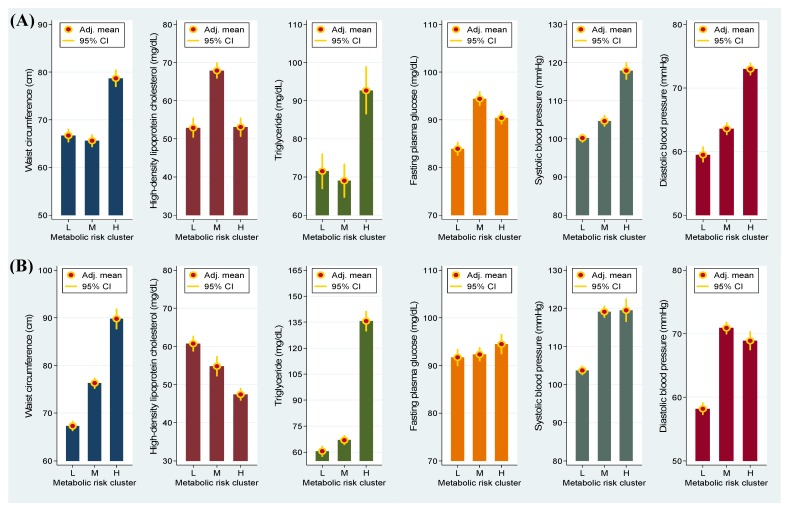
Adjusted (adj.) mean and 95% confidence intervals (CI) of metabolic syndrome components associated with low (L), median (M) and high (H) metabolic risk clusters in girls (**A**) and in boys (**B**).

As shown in Figure 1, boys at a high metabolic risk exhibited greater WC and TG, FPG, SBP and DBP levels than did those at a low metabolic risk. Similarly, a higher level of these four MetS components and a lower concentration of HDL-C were observed in girls at a high metabolic risk compared with those at a low metabolic risk. These results indicated that the data-driven risk clusters were suitable for determining the risk of developing adolescent MetS.

Table 4 shows the effect of SSB intake on metabolic risk clusters among adolescents. Increased SSB consumption was determined to be associated with a high metabolic risk cluster in both sexes (*P*_trend_ < 0.038). Adolescents who drank >500 mL of SSBs per day exhibited a 1.9-fold (95% CIs: 1.1-3.1) and a 2.7-fold (95% CIs: 1.3-5.7) risk of being classified as having a high overall metabolic risk in girls and boys, respectively.

**Table 1:** Distributions of demographic factors, physical activity and energy intake associated with sugar-sweetened beverage consumption (mL/day) among adolescents in Taiwan.

Factors	Girls				Boys			
	Nonintake	1–500	>500	*p* ^3^	Nonintake	1-500	>500	*p* ^3^
**Study subjects (no.) ^1^**	196	961	242		120	802	406	
**Population distribution ^2^**								
**Consumer pattern (%)**	15.6	65.9	18.5		8.8	59.1	32.1	
**Age, years (Mean ± SE)**	13.6 ± 0.1	13.5 ± 0.1	13.7 ± 0.1	0.574	13.6 ± 0.1	13.5 ± 0.1	13.7 ± 0.1	0.058
**Area (%)**								
Kaohsiung city	19.4	63.2	17.4	0.033	9.3	57.8	32.9	0.746
Pingtung county	10.6	68.3	21.2		7.7	60.6	31.7	
Taitung county	11.0	74.2	14.8		9.2	62.8	28.0	
**Physical activity, MET-min/week (%)**								
<952.4	16.1	63.1	20.8	0.324	9.4	54.8	35.8	0.106
952.5–2140.4	16.7	68.0	15.3		11.5	55.8	32.7	
≥2140.5	12.0	65.9	22.1		6.0	63.9	30.1	
**Total calories, kcal/day (Mean ± SE)**	1835.3 ± 61.3	1903.4 ± 29.9	2044.8 ± 45.3	<0.001	2083.7 ± 60.8	2151.5 ± 39.9	2448.2 ± 67.2	<0.001

^1^ Raw number of study samples (the number that is not adjusted for sample survey design); ^2^ Data was presented adjusted for sample weight and complex sample design; ^3^*p* for associations between sugar-sweetened beverage consumption and the factors investigated.

**Table 2:** Multivariate-adjusted means (aMean) ^1,2^ and regression coefficients (adj. β) ^1,3^ of cardiometabolic risk factors associated with sugar-sweetened beverage consumption (mL/day) among adolescents in Taiwan.

Factors	Girls									Boys								
	Nonintake		1–500		>500					Nonintake		1–500		>500				
	aMean	SE	aMean	SE	aMean	SE	adj. β	SE	*p*_trend_	aMean	SE	aMean	SE	aMean	SE	adj. β	SE	*p*_trend_
**Waist circumference, cm**	68.0	0.9	69.4	0.7	71.1 *	0.8	1.5	0.6	0.011	72.6	0.9	75.0 *	0.6	76.3 *	0.9	1.6	0.7	0.039
**HDL cholesterol, mg/dL**	58.8	1.3	59.0	1.2	57.6	1.6	−0.6	0.9	0.528	56.0	1.3	56.3	0.7	54.9	1.6	−0.9	1.0	0.401
**Triglyceride, mg/dL**	74.4	2.3	76.0	1.9	79.3	3.2	2.5	1.7	0.160	70.4	3.2	78.4 *	1.6	78.6 *	2.8	2.6	2.4	0.278
**Plasma glucose, mg/dL**	89.1	0.9	89.6	0.7	90.6	0.9	0.7	0.5	0.119	92.1	1.0	93.1	0.6	91.4	1.0	−0.9	0.6	0.167
**SBP, mmHg**	106.2	1.3	106.5	0.6	107.7	1.2	0.8	0.6	0.174	111.9	1.3	112.2	0.9	114.3	0.6	1.6	0.7	0.043
**DBP, mmHg**	65.2	0.8	64.5	0.3	65.3	0.7	0.1	0.5	0.890	65.5	1.1	65.3	0.6	65.1	0.6	−0.2	0.6	0.753
Total cholesterol, mg/dL	161.1	3.9	164.2	2.4	166.1	3.7	2.5	2.2	0.274	154.2	2.4	158.1	1.5	156.5	4.4	0.1	2.8	0.984
Hip circumference, cm	89.8	0.6	90.1	0.4	92.3 *	0.7	1.3	0.4	0.007	90.2	0.8	91.7	0.5	93.8 *	0.7	2.0	0.6	0.002
Body fat, %	26.1	0.5	26.7	0.3	28.5 *	0.5	1.2	0.4	0.005	17.7	1.0	19.8 *	0.5	20.8 *	0.6	1.3	0.6	0.030
Body adiposity index, %	27.6	0.3	27.9	0.2	29.1 *	0.3	0.8	0.2	0.001	24.9	0.4	26.2 *	0.2	26.7 *	0.3	0.8	0.3	0.005
Body mass index, kg/m^2^	20.3	0.3	20.7	0.2	21.5 *	0.3	0.6	0.2	0.007	21.0	0.4	22.0 *	0.2	22.7 *	0.3	0.8	0.3	0.009

Abbreviations: * *p* < 0.05; HDL, high-density lipoprotein; SBP, systolic blood pressure; DBP, diastolic blood pressure; adj., adjusted. ^1^ Models were adjusted for study area, age, physical activity, total calories, the intake of meat, fruit, fried food, food with jelly/honey, alcohol drinking and cigarette smoking; ^2^ Adjusted mean displays the estimated prediction when the covariates were set as mean values; ^3^ Adjused regression coefficients were estimated for a linear dose-response effect of sugar-sweetened beverage consumption.

**Table 3:** Prevalence rates and adjusted odds ratios (aOR) ^1^ of metabolic syndrome (MetS) defined by IDF and Cook criteria associated with sugar-sweetened beverage consumption (mL/day) among adolescents in Taiwan.

Factors	Girls								Boys							
	Prevalence, %			1–500 *vs*. NI		>500 *vs*. NI			Prevalence, %			1–500 *vs*. NI		>500 *vs*. NI		
	NI	1–500	>500	aOR	(95% CI)	aOR	(95% CI)	*p*_trend_	NI	1–500	>500	aOR	(95% CI)	aOR	(95% CI)	*p*_trend_
**IDF criteria**																
Large WC	8.2	13.4	22.7	2.0	(0.99–3.9)	4.1	(1.9–8.8)	<0.001	7.4	12.1	18.7	2.0	(0.8–5.1)	3.0	(1.2–7.6)	0.011
Low HDL-C	5.2	9.1	11.2	1.8	(0.8–4.4)	2.1	(0.7–6.7)	0.194	4.3	9.5	13.5	2.4	(0.7–8.1)	3.9	(0.9–16.4)	0.067
Elevated TG	2.3	4.0	4.6	1.9	(0.8–4.4)	2.1	(0.7–6.8)	0.197	3.9	5.5	7.1	1.3	(0.5–3.7)	1.7	(0.6–5.1)	0.289
Increased FPG	9.0	10.8	11.8	1.3	(0.6–2.5)	1.5	(0.9–2.4)	0.203	17.2	19.4	17.3	1.2	(0.6–2.4)	1.1	(0.6–2.2)	0.908
High BP	3.6	4.3	4.9	1.2	(0.3–4.3)	1.5	(0.5–5.2)	0.423	12.9	10.8	15.5	0.8	(0.3–2.3)	1.2	(0.5–3.1)	0.244
Component (c) no.																
1c	21.8	29.6	27.7						23.2	26.6	27.8					
2c	**3.3**	**4.3**	**10.6**						**10.4**	**9.1**	**13.1**					
3c	**0.0**	**1.1**	**2.1**						**0.6**	**2.2**	**5.5**					
**MetS (WC + ≥2c)**	**0.0**	**1.1**	**2.1**	**3.8 ^2^**	**(0.6–∞)**	**6.5 ^2^**	**(0.9–∞)**	**0.049 ^2^**	**0.6**	**3.5**	**5.4**	**6.9**	**(0.7–63.2)**	**10.3**	**(1.2–90.2)**	**0.007**
**Cook criteria**																
Large WC	6.1	10.7	10.5	2.1	(1.0–4.4)	2.3	(1.1–4.8)	0.014	5.9	9.7	14.7	2.1	(0.9–5.0)	3.1	(1.4–6.9)	0.008
Low HDL-C	5.2	9.1	11.3	1.8	(0.8–4.4)	2.2	(0.7–6.7)	0.184	4.9	9.5	13.5	2.1	(0.7–6.7)	3.4	(0.8–14.0)	0.083
Elevated TG	9.2	12.9	15.0	1.6	(0.9–2.9)	1.9	(0.96–3.7)	0.065	9.7	16.3	18.9	1.9	(0.9–4.0)	2.3	(1.1–5.0)	0.045
High FPG	1.4	1.9	1.8	1.9	(0.7–4.9)	1.6	(0.2–10.8)	0.690	0.0	1.9	1.5	3.6 ^2^	(0.6–-∞)	2.0 ^2^	(0.3–∞)	1.000 ^2^
Raised BP	12.5	16.2	23.0	1.3	(0.8–2.1)	2.1	(1.3–3.7)	0.015	18.0	17.3	22.0	0.9	(0.4–1.9)	1.2	(0.6–2.6)	0.266
Component (c) no.																
1c	22.5	29.1	21.1						26.6	24.8	31.5					
2c	**5.1**	**8.0**	**14.7**						**4.4**	**8.3**	**11.6**					
3c	**0.6**	**1.7**	**2.5**						**1.0**	**3.1**	**4.5**					
**MetS (≥3c)**	**0.6**	**1.9**	**3.4**	**3.2**	**(0.6–17.8)**	**5.9**	**(0.8–43.8)**	**0.085**	**1.0**	**4.1**	**5.1**	**4.9**	**(1.1–21.1)**	**5.1**	**(1.01–25.5)**	**0.251**

Abbreviations: NI, nonintake; IDF, International Diabetes Federation; WC, waist circumference; HDL-C, high-density lipoprotein cholesterol; TG, triglyceride; FPG, fasting plasma glucose; BP, blood pressure; ^1^ ORs were adjusted for study area, age, physical activity, total calories, the intake of meat, fruit, fried food, food with jelly/honey, alcohol drinking and cigarette smoking; ^2^ ORs were calculated using the median unbiased estimates with the aid of exact logistic regression.

**Table 4:** Prevalence rates and adjusted odds ratios (aOR) ^1^ of high metabolic risk cluster associated with sugar-sweetened beverage consumption (mL/day) among adolescents in Taiwan.

Factors	Girls								Boys							
	Prevalence, %			1–500 *vs*. NI		>500 *vs*. NI			Prevalence, %			1–500 *vs*. NI		>500 *vs*. NI		
	NI	1–500	>500	aOR	(95% CI)	aOR	(95% CI)	*p*_trend_	NI	1–500	>500	aOR	(95% CI)	aOR	(95% CI)	*p*_trend_
**Metabolic risk cluster ^2^**																
Low	35.2	34.7	33.5	1.0		1.0			42.8	43.6	37.1	1.0		1.0		
Median	42.0	38.5	34.5	1.2	(0.8–1.8)	1.2	(0.7–2.2)	0.433	47.4	36.6	40.9	0.8	(0.5–1.3)	1.0	(0.6–1.6)	0.530
High	22.8	26.9	32.1	1.4	(0.9–2.3)	1.9	(1.1–3.1)	0.020	9.8	19.8	22.1	2.2	(1.0–4.8)	2.7	(1.3–5.7)	0.038

Abbreviations: NI, nonintake; ^1^ ORs were adjusted for study area, age, physical activity, total calories, the intake of meat, fruit, fried food, food with jelly/honey, alcohol drinking and cigarette smoking; risk cluster groups were derived from a two-step cluster analysis.

## 4. Discussion

This study presents data indicating that increased SSB intake is associated with an increased WC, HC, total cholesterol, body fat, BAI and BMI among schoolchildren. In boys, the consumption of a high amount of SSBs exhibited a significant effect on development of MetS; adolescents who ingested >500 mL of SSBs per day were likely to exhibit a high overall metabolic risk.

Several mechanisms explaining the link between SSB consumption and weight gain have been suggested [18,19,20]. Although satiety responses vary according to race, sex and body weight, liquid carbohydrates generally provide a lower level of satiety than do solid carbohydrates [18,19]. Energy obtained from liquid sources that provide low levels of satiety has been observed to result in poor compensation by subsequent energy intake, thereby inducing an increase in total energy intake [19,20]. In addition, adult diets containing SSBs have been linked to an increase in self-selected daily energy intake [20]. Recently, a systematic review and meta-analysis reported that the intake of SSBs is clearly associated with increased energy intake and body weight [31]. The results obtained in this investigation support this argument.

The association between SSB intake and adiposity has been extensively assessed in observational studies, experimental trials and meta-analyses [20,32,33]. The majority of well-designed observational studies have reported that the consumption of several types of SSB has detrimental effects on adiposity and obesity [20,33]. Numerous findings of studies that were designed to reduce and increase SSB consumption have supported a causal relationship between SSB intake and weight gain [32,33]. Taking all concerns regarding risk evaluation into account, a meta-analysis study identified a significant 0.08-unit change in BMI associated with each 355 mL of SSB intake [32]. In this study, we observed that the BMI values increased as SSB consumption increased in both girls and boys. The phenotypes of weight gain were observed in WC, HC and body fat. Among these, a large WC was the criterion required for an IDF MetS diagnosis [7]. Compared with nondrinkers, the results indicated that boys and girls who drank a high amount of SSBs exhibited 4.6% and 5.1% significantly higher WC, respectively (68.0 cm boosted to 71.1 cm in girls and 72.6 cm boosted to 76.3 cm in boys). A recent prospective investigation reported compatible results; specifically, compared to the lowest tertile, a 2.3%-4.2% notably higher WC occurred among teens aged 14-17 years who were in the highest tertile for amount of SSBs consumed [3]. In two U.S. National Health and Nutrition Examination Survey studies conducted in 1999-2004, in which data obtained from young children (3-11 years) and adolescents (12-19 years) were examined, a significant association between SSB consumption and WC was identified among the 9-11- and 12-19-year-old subgroups [34,35]. These findings emphasized that WC is a central MetS component among adolescents.

The present study indicated that boys who ingested 1-500 and >500 mL of SSBs per day exhibited TG levels that were 8.0 and 8.2 mg/dL higher than those of nondrinkers, respectively. Comparable findings have been observed among female teenagers aged 12-19 years (each additional intake of 250 g/day of SSBs was related to a 2.3 mg/dL increase in TG levels) according to a nationwide survey conducted in the United States, although a nonsignificant increase in TG was observed among male teenagers [35]. In an adolescent cohort study conducted in Australia, a BMI-independent link between high quantities of SSB intake and TG concentrations was recognized among both girls and boys, indicating that the effect of high SSB consumption on lipid accumulation may occur through various mechanisms, excluding excess weight [3]. Alternatively, elevated serum TG levels have been associated with a high intake of fructose-rich SSBs in adolescents [36].

Research findings concerning the effects of SSB consumption on adolescent blood pressure have been inconclusive, although an adverse effect on SBP has been suggested [3,35,37]. A large-scale cross-sectional study demonstrated that adolescents aged 12-18 years who had consumed a high amount of SSBs exhibited a 2-mmHg (95% CIs: 1 to 2 mmHg) higher SBP compared with nondrinkers [37]. A 0.16-mmHg elevated SBP associated with consuming one serving of SSB was also observed among nationally representative teenagers aged 12-19 years in the U.S. [35]. In a prospective study of adolescents, a 1.7% higher SBP was observed in girls who exhibited high SSB consumption; however, the effect was not significant in boys [3]. The present findings support the detrimental effect on SBP, because a positive dose-response relationship between SSB intake and SBP was identified among boys. In assessing study findings obtained from five electronic databases, a comprehensive systemic review indicated that SSB consumption is linked to increased BP and an increased incidence of hypertension [38].

Although the IDF criteria and Cook *et al*. criteria for diagnosing adolescent MetS are slightly different [7,26], this study revealed that the prevalence of MetS increases as the intake of SSBs increases in both girls and boys. Because MetS prevalence is relatively lower in adolescents than in adults, risk assessments of pediatric MetS related to SSB intake that have been conducted in prior studies have focused on MetS components [34,35,37]. The present results indicated that boys who consumed a high amount of SSB exhibited a 5.1- to 10.3-fold risk of developing MetS, even if the prevalence of this metabolic disorder was low. In the two-step cluster analysis, which was used to determine the nature clusters of adolescents with similar levels of metabolic risk, adolescents who drank a high amount of SSBs were determined to have a 1.9- to 2.7-fold risk of being included in a high metabolic risk cluster. Recently, a longitudinal study provided comparable results; specifically, a high SSB intake was dose-dependently linked to a high prospective metabolic risk in girls [3]. Because the development of MetS in childhood is a significantly predictor of adult MetS, type 2 diabetes and cardiovascular disease 25 to 30 years later [11,12], public health advocates must become aware of the problems associated with SSB intake and the likelihood of developing MetS in adolescents.

The major strength of this study is that several crucial confounding variables were adjusted for all of the assessments. A high metabolic risk group derived from the two-step cluster analysis was used to facilitate the risk evaluation of SSB intake in an adolescent population with a low prevalence of MetS. Alternatively, the results of this investigation must be interpreted carefully because SSB consumption, anthropometric parameters and biochemical outcomes were measured only once. We were unable to provide causal explanations because of the cross-sectional nature of the findings.

## 5. Conclusions

In conclusion, a high SSB intake is associated with adolescent MetS among boys but not girls in Taiwan.

## Abbreviations

aOR: adjusted odds ratio
BAI: body adiposity index
BMI: body mass index
CI: confidence interval
DBP: diastolic blood pressure
FPG: fasting plasma glucose
HC: hip circumference
HDL-C: high-density lipoprotein cholesterol
IDF: International Diabetes Federation
LDL-C: low-density lipoprotein cholesterol
MET: metabolic equivalent task
MetS: metabolic syndrome
SSB: sugar-sweetened beverage
SBP: systolic blood pressure
TG: triglycerides
WC: waist circumference
